# Intuitive Spatial Tactile Feedback for Better Awareness about Robot Trajectory during Human–Robot Collaboration

**DOI:** 10.3390/s21175748

**Published:** 2021-08-26

**Authors:** Stefan Grushko, Aleš Vysocký, Dominik Heczko, Zdenko Bobovský

**Affiliations:** Department of Robotics, Faculty of Mechanical Engineering, VSB-TU Ostrava, 70800 Ostrava, Czech Republic; ales.vysocky@vsb.cz (A.V.); dominik.heczko@vsb.cz (D.H.); zdenko.bobovsky@vsb.cz (Z.B.)

**Keywords:** human–robot collaboration, human–robot interaction, mutual awareness, haptic feedback device, human–machine interface, spatial tactile feedback

## Abstract

In this work, we extend the previously proposed approach of improving mutual perception during human–robot collaboration by communicating the robot’s motion intentions and status to a human worker using hand-worn haptic feedback devices. The improvement is presented by introducing spatial tactile feedback, which provides the human worker with more intuitive information about the currently planned robot’s trajectory, given its spatial configuration. The enhanced feedback devices communicate directional information through activation of six tactors spatially organised to represent an orthogonal coordinate frame: the vibration activates on the side of the feedback device that is closest to the future path of the robot. To test the effectiveness of the improved human–machine interface, two user studies were prepared and conducted. The first study aimed to quantitatively evaluate the ease of differentiating activation of individual tactors of the notification devices. The second user study aimed to assess the overall usability of the enhanced notification mode for improving human awareness about the planned trajectory of a robot. The results of the first experiment allowed to identify the tactors for which vibration intensity was most often confused by users. The results of the second experiment showed that the enhanced notification system allowed the participants to complete the task faster and, in general, improved user awareness of the robot’s movement plan, according to both objective and subjective data. Moreover, the majority of participants (82%) favoured the improved notification system over its previous non-directional version and vision-based inspection.

## 1. Introduction

Collaborative robot applications bring new possibilities for the automation of manufacturing processes and better adaptability of the work task in a low structured environment through human intelligence, perception, and decision-making ability [1]. As a result, the robot task and production technology can be significantly simplified since the human worker can easily do the cognitively more demanding part. However, most of the modern collaborative industrial applications are limited by the fact that neither collaborative side is fully aware of the partner: the human operator may not see the robot movement due to own engagement in the work process, and the collaborative robot simply has no means of knowing the position of the operator. This fact may manifest in possible collisions, which are, however, relatively safe, as the collaborative robots are lightweight and highly sensitive to the impacts. Nevertheless, the perspective of active collision avoidance [2,3,4,5] may represent an efficiency boost to the work process, especially in work tasks with high variability. A collaborative workspace is a good example of a dynamic environment, where the human worker represents an element with the highest variability. Enabling a robot system to monitor its surroundings using range imaging sensors and enabling it to adapt trajectories to the motions of the operator partially diminish the risk of collision. Still, it can be expected that improving human awareness about the robot motion plans would translate into even better performance [2], as this could allow the worker to better plan own actions, avoid robot interference, and be more confident while working with the robot. Moreover, in some cases, the position of the operator may be completely blocking the robot from performing the task, and the user’s unawareness may lead to the lower overall productivity of the workspace or failed technological processes, for instance, when the robot task is strictly limited in time. Mutual understanding and awareness between human and robot are thus a substantial aspect of achieving good efficiency and ergonomics in task execution.

Better user awareness can be provided by utilising notification devices (human–machine interfaces, HMIs) that allow to understand motion plans and status of the robot. In order to convey information, these systems may utilise the primary sensory modalities of a human: vision (monitors [6], light projectors [7,8], mixed reality devices [9,10,11]), hearing (sound alerts and speech notifications [2,12]), touch (tactile feedback devices [4,13,14,15]). Touch modality represents a robust and direct way of transferring information to the user, making it suitable to convey information to workers in industrial environments, where visual and auditory modalities might be busy or blocked. Haptic feedback devices typically utilise vibration stimuli to convey information to the user. Their implementations often utilise vibration motors for providing feedback to the user as they allow continuous stimulation for unlimited time. However, other implementations utilising pneumatic chambers [16], actuated sliders and mechanisms [17,18] are known. The particular utilised activation of the tactile actuators and its consistency with the desired reaction of the user is also an important factor influencing the intuitiveness of the provided stimuli, as demonstrated in the study performed by Scalera et al. [19], in which the researchers compared user performance in a joystick control task with stimuli delivered either using tactors with attractive (move toward the vibration) or repulsive prompts (move away from the vibration). Due to the fact that these devices must be in direct contact with the skin to activate mechanoreceptors, the devices are often implemented as wearable equipment. This ensures that notifications are reliably transmitted to the user regardless of the circumstances, unlike graphical clues that may be out of the view angle of the user. P. Barros et al. [15] performed a set of tests using the simulation model of the teleoperated robot and enabled the users with tactile feedback that notified them about the actual collisions of the robot with the surroundings. Vibration device can also be used during the control of industrial robot notifying the user about approaching singularities, joint limits or commencing the next phase of the manufacturing process [4].

In our previous work [20], we presented a concept of a novel wearable haptic notification system for informing the human operator about the robot’s status, its currently planned trajectory and the space that will be occupied by the robot during the movement. We also introduced an initial implementation of the described system, which consisted of two wearable glove-integrated haptic devices which provided the user with vibration feedback of intensity dependant on the distance between the hand and the future trajectory of the robot. A user study was performed in order to assess the usability of the system. During the user study, a possibility to extend the vibration alerts and provide the user with more intuitive orientation-dependent tactile feedback was noticed.

In the present work, we further extend the developed system and present a principle of spatial notifications. The main change consists in a new notification mode for the hand-worn haptic devices, in which the vibration alerts are provided to the user using a set of spatially organised tactors, where the intensity of the vibration of each tactor depends both on the relative position and orientation of the device and future trajectory of the robot. It is anticipated that this enhancement will further improve the user’s awareness regarding the robot’s trajectory by providing additional information about the direction in which a possible collision may occur. Pattern-based tactile feedback (also tactile displays) approaches have been applied by several research groups in order to assist visually impaired people in tasks of spatial navigation [21,22], accessing digital content [23,24], movement guiding [25], interaction with virtual objects [26]. S. Williams and A. Okamura have demonstrated [27] a wearable vibrotactile display for communicating encoded messages to the user and investigated the influences of different modes of the proposed system on the ability of the users to accurately identify the vibrotactile stimuli. M. Aggravi et al. [28] implemented a solution for guiding the hand of a human user using a vibrotactile haptic device placed on the user’s forearm. A similar solution was implemented in the study of S. Scheggi et al. [29,30], in which a mobile robot had the task of guiding a human (possibly sightless) from an initial to the desired target position through a cluttered corridor by only interacting with the human via bracelet with three embedded vibration motors. K. Yatani et al. have presented [31] a handheld system consisting of an array of vibration tactors attached to a smartphone sleeve for representing geographical information. The tactors’ activation patterns of the device offered high-level information about the distance and direction towards a destination during navigation tasks. However, to the best of our knowledge, the system presented in our work is the first example of use of the spatial tactile feedback for informing the user about the currently planned trajectory of the robot.

## 2. Materials and Methods

### 2.1. Improved Mutual Awareness for Human–Robot Collaboration

**Figure 1:**
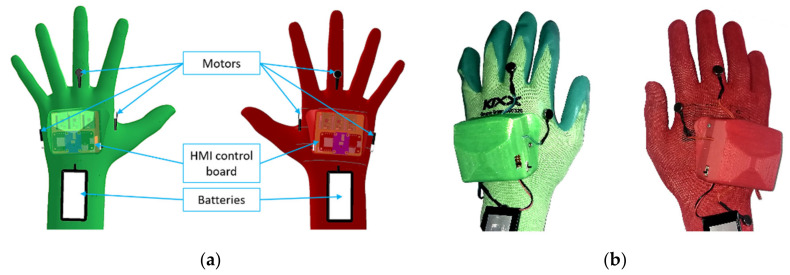
Prototype of HMI: (**a**) 3D model of HMI prototype, HMI cover is set as translucent in order to visualise the internal placement of the components; (**b**) implemented prototype.

In the previous work, we proposed a concept of a shared collaborative workspace where the robot can adapt its movement to avoid collision with human workers. The workspace is monitored by multiple RGB-D sensors, and data provided by these sensors allow to construct a map of the robot’s surroundings and obstacles. At each step of the task execution, the robot creates a collision-free motion plan according to the currently available free space. If during the execution of the planned movement there is a change in the environment and the movement can no longer be completed due to possible collisions with obstacles, the robot can create a new motion plan (active collision avoidance). The human–robot collaboration is enhanced by introducing haptic feedback devices (HMIs), whose task is to reliably notify the human worker about the currently planned robot’s trajectory and changes in its status. With regard to involvement in the work process, the hands are the parts of the body that are most often present in the shared workspace. For this reason, it was decided to develop a haptic HMI in the form of a compact device attached to the dorsal side of a work glove providing vibrational feedback to the user’s palm (see Figure 1). The human worker is equipped with two haptic feedback devices placed on each hand. It was also decided to use distinctive colours for HMIs: the left HMI is green, and the right HMI is red. These colours simplify the task of hand tracking and determining the side of the hand.

**Figure 2:**
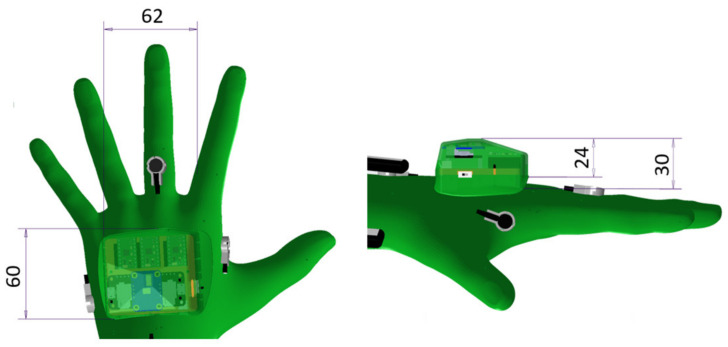
HMI hardware design - main dimensions.

The design ensures that the HMIs do not bind the user during manual work, as it does not restrict finger movements (see Figure 2). The battery is placed on the user’s wrist, which also improves the overall ergonomics of the device. The total weight of one HMI is 150 g. Each glove is equipped with six ERM (eccentric rotating mass) vibration motors, which provide haptic feedback. The vibration motors are controlled by three compact dual DC motor PWM drivers. The new version of the notification device is equipped with a 9-axis IMU sensor, that is used to obtain the orientation of HMIs in the workspace.

**Figure 3:**
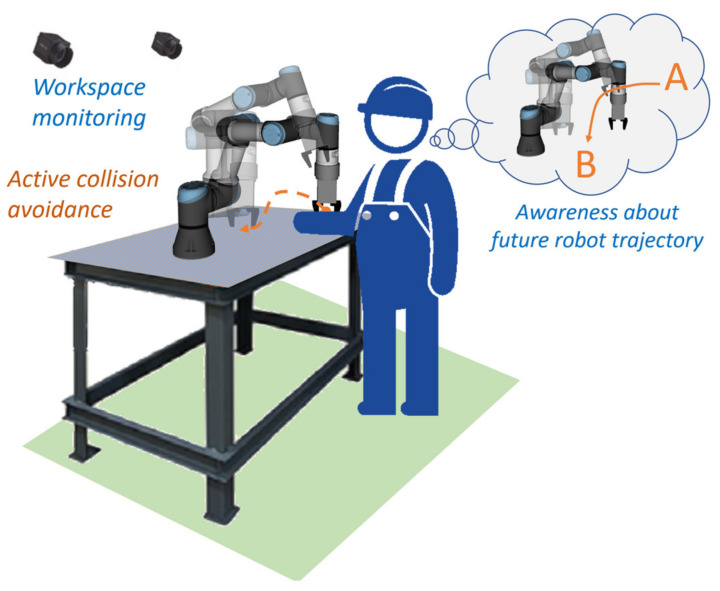
The concept of improved HRC combines principles of active collision avoidance with an increased awareness provided by the wearable haptic feedback device.

The notification system utilises three types of notifications to inform the operator about the status of the robot and its trajectory (see Figure 3): distance notification, replanning notification, and inaccessible goal notification.

**Figure 4:**
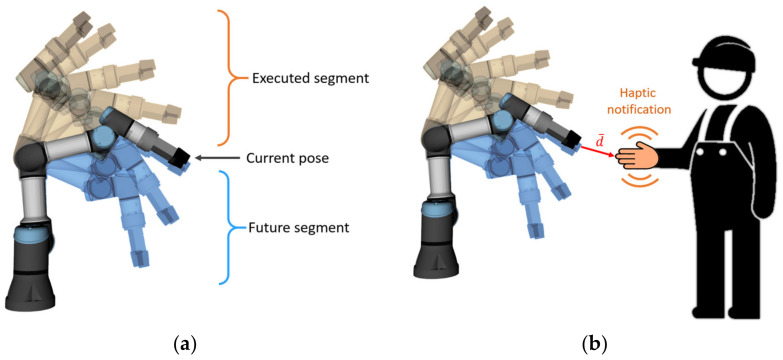
(**a**) Trajectory execution: already executed segment of trajectory, current state, and future segment (planned trajectory segment yet to be executed); (**b**) human worker equipped with haptic feedback device, which provides vibration alert about the proximity to the future trajectory of the robot; collision vector is denoted as $\vec{d}$.

The distance notification provides a continuous vibration alert to the user about the proximity to the currently planned trajectory of the robot. The future trajectory segment is defined as the part of the feasible trajectory that yet has not been executed (see Figure 4a). The closer the worker’s hand (equipped with HMI) approaches the future segment of the trajectory, the stronger the vibration provided by the device (see Figure 4b). The length of the vector between the nearest points of HMI and the robot body in all timesteps of the future trajectory (hereinafter “collision vector”, $\vec{d}$) is considered to be the distance, which is used to calculate the vibration intensity. There is also an upper limit of the distance at which HMIs provide feedback—reaction distance *d_r_*. This ensures that the worker receives an alert only if current actions may interfere with the robot trajectory.

In the previous work, the distance notifications consisted only in simultaneous activation of all tactors located on the corresponding HMI with the same intensity (hereinafter simultaneous motor activation mode, SMAM). The distance-dependant vibration intensity of the distance notification is then calculated according to (1).

(1) $$v_{d}=\frac{(v_{p,max}-v_{p,min})*\left(d_{r}-\left|\vec{d}\right|\right)\;}{d_{r}+v_{p,min}},$$

where $v_{d}$ calculated vibration intensity for distance notification,

$v_{p,max}$—maximum vibration intensity for distance notification,
$v_{p,min}$—minimum vibration intensity for distance notification,
$d_{r}$—reaction distance—distance threshold at which the distance notification is activated,
$\vec{d}$—current collision vector (see Figure 4).

The reaction distance $d_{r}$ was set to 15 cm as it was found the most convenient for the users during preliminary tests. For SMAM, the calculated intensity $v_{d}$ is then applied to all tactors of the corresponding haptic device (by sending motors’ speed change requests via BLE communication).

Apart from distance notification, there are two more types of notification, the purpose of which is to notify the user about the status of the robot. When a person, despite a warning, interferes with the currently planned trajectory, the robot attempts to find a new feasible path to the goal position and continues the activity. Every time a new trajectory is planned as a result of an environment change, both feedback devices use strong vibration notification (hereinafter “replanning notification”, this notification has 0.3 s duration) to draw the attention of the human worker and to indicate that the robot has detected an environment change and has replanned its movement. If no feasible path to the target has been found, both feedback devices also provide a strong vibration alert (hereinafter “inaccessible goal notification”).

### 2.2. Improved Haptic Notification Devices

In this work, we further extend the previously presented concept of the haptic notifications for informing the user about the planned trajectory of the robot by introducing the proportional orientation-dependent (spatial) tactile notifications.

This concept was implemented in the form of a new mode for the HMI devices: Directional Motor Activation Mode (hereinafter DMAM). The core principle of DMAM is following: the vibration activates on the side of the HMI glove that is closest to the future path of the robot (repulsive prompt to the user). This particular activation was chosen due to the results of the preliminary tests on a small number of subjects, which has demonstrated that the vibration stimuli provided by the individual tactors of the gloves are intuitively perceived by the users as repulsive; however, a standalone investigation of the effects of different tactors’ activation types is required in future. If the corresponding hand starts to move away from the robot’s trajectory, the vibration will decrease until it disappears completely. Thus, in real conditions, in order to prevent a collision, one must move the hand in the opposite direction to the percept vibration.

**Figure 5:**
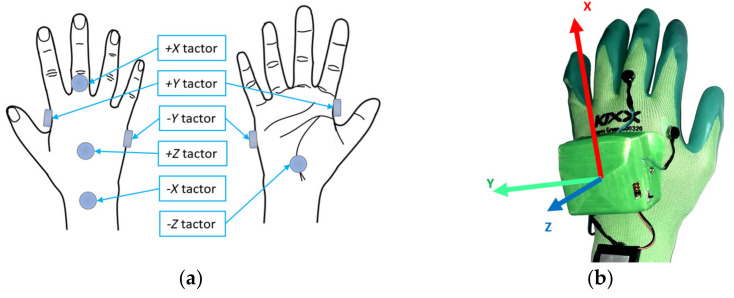
Tactors placement around the hand: (**a**) function of each tactor (positive and negative directions of X, Y, Z axes); (**b**) spatial organisation represents a coordinate frame.

In this mode, the distance notification provides the user with additional information about the direction to the nearest point of the robot body in the future trajectory. The vibration intensity of each motor located on the corresponding HMI glove is dependent on both the spatial configuration of the collision vector $\vec{d}$ and its magnitude, as well as the relative orientation of the corresponding HMI. Total activation intensity is distributed among adjacent motors according to the relative spatial orientation of the corresponding HMI and collision vector $\vec{d}$: the motors that are closer to the future segment of the robot trajectory will vibrate more intensively. The new versions of HMI are equipped with IMU sensors in order to obtain the orientation of the devices. DMAM was developed assuming that directional feedback may be more intuitive for the users and communicate more information to them. In order to communicate information about direction through activation of the tactors fixed to the glove, their spatial organisation was updated to represent an orthogonal coordinate frame (see Figure 5). This change was done to improve the intuitiveness of the devices: it is assumed that the user will be able to both recognise the intensity of vibration of individual tactors and the communicated information about the direction.

**Figure 6:**
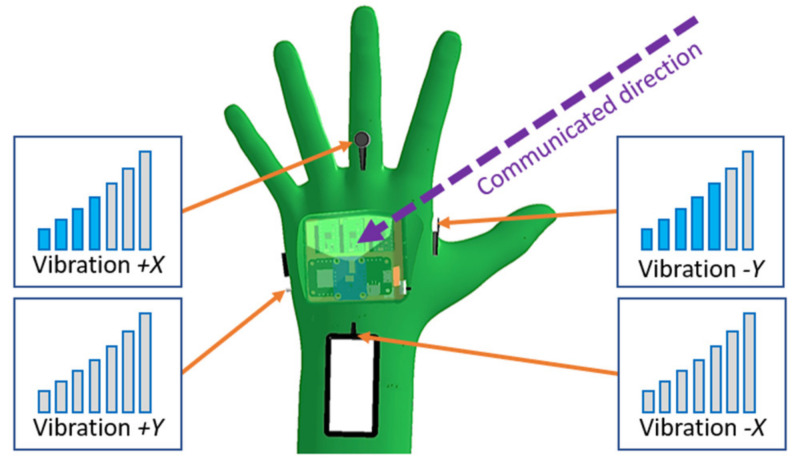
In directional feedback mode, HMI notifies the user about the direction of a possible collision through a difference in activation of tactors located around the hand.

An arbitrary vector in this configuration is represented by activation of multiple adjacent motors with different vibration intensities (see Figure 6). 

**Figure 7:**
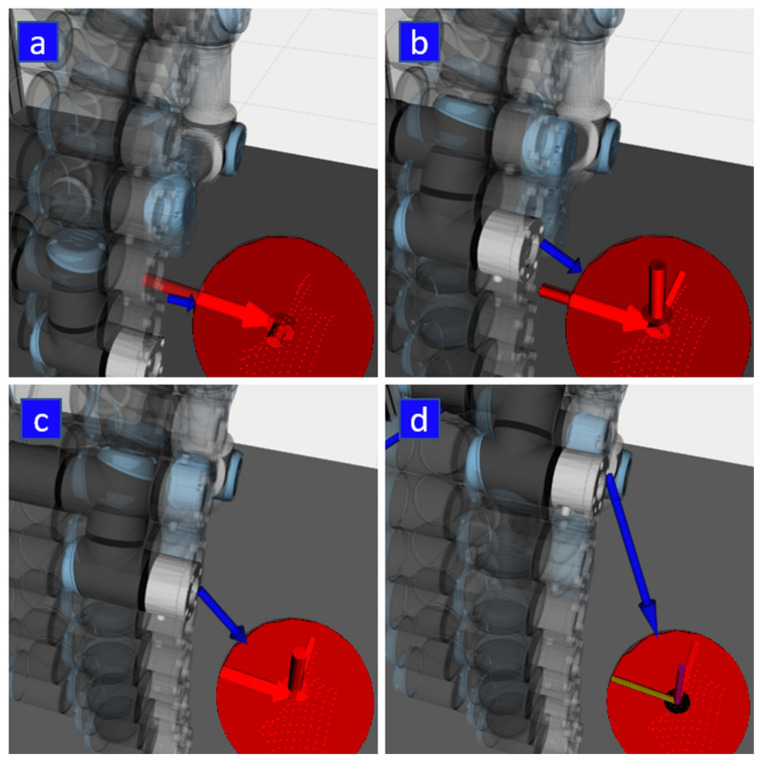
Visualisation of the vibration intensity of the tactors, lengths of the red vectors depict the intensity of vibration of right HMI tactors, which are dependent on the magnitude of the collision vector (blue arrow) and the relative orientation of the corresponding HMI. The robot is moving from the bottom (**a**) through intermediate position (**b**) to the upper position (**c**) until no vibration is provided (**d**) since the magnitude of the collision vector is higher than the reaction distance. The red sphere represents right HMI; red points represent HMI point cloud; HMI axes are shown by XYZ frame (**d**).

However, due to the difference in the sensitivity of skin regions around the hand [32], it is anticipated that more research will be needed to find optimal placement for the motors since the perceived vibration intensity may vary across the currently chosen locations of the motors. For DMAM, the calculated intensity $v_{d}$ is considered to be the total vibration intensity, which is further distributed between the HMI tactors according to the current space configuration of the collision vector $\vec{d}$ (see Figure 7).

Rotation matrix $R_{world\to HMI}$, which describes the orientation of HMI in world frame (obtained from IMU data), is utilised to transform the normalised collision vector $\vec{d}$ into HMI frame. The vibration intensity vector ${\vec{s}}_{d}$ is then calculated according to (2):

(2) $${\vec{s}}_{d}=R_{world\to HMI}\;\cdot \;\;\frac{\vec{d}}{\left|\vec{d}\right|}\;\cdot \;v_{d}$$

Total vibration intensity $v_{d}$ is then distributed to the corresponding tactors by simply picking the absolute values of the corresponding component of the vibration intensity vector ${\vec{s}}_{d}$. The actual intensity values for tactors belonging to the same axis are defined by (3) and (4):

(3) $$v_{+x}=\left\{\begin{array}{c} {\vec{s}}_{d,x},\;\;\mathrm{if}\;{\vec{s}}_{d,x}>0\\ \;0,\;\;\;\;\;\;\;\;\;\;\;\;if\;{\vec{s}}_{d,x}<0 \end{array}\right.$$

(4) $$v_{-x}=\left\{\begin{array}{c} \;\;0,\;\;\;\;\;\;\;\;\;\;\;\;\;\;\;if\;{\vec{s}}_{d,x}>0\\ \;\left|{\vec{s}}_{d,\;x}\right|,\;\;\;\;\;\;\;\;\;\;\;\;\;\;\;\;if\;{\vec{s}}_{d,x}<0 \end{array}\right.$$

where $v_{+x}$—vibration intensity for the tactor representing the positive direction of *X*-axis,

$v_{-x}\;$≤ vibration intensity for the tactor representing the negative direction of the *X*-axis.

Apart from distance notification, replanning and inaccessible goal notifications work the same way as in SMAM.

Proportional activation of tactors was used as an alternative to pattern approaches [33,34] as it was found more convenient and intuitive for the users during preliminary tests. The delays caused by the application of pattern-based activation of tactors was concluded to be inappropriate for the dynamic environment of a collaborative workspace.

Vibration intensities were handpicked for both modes to make the haptic feedback unobtrusive during its activation. Activation intensities for individual notifications are shown in Table 1, where the intensity of vibration is represented in the range 0–100% of PWM duty cycle, where 0% represents no vibration, 100% represents the maximum attainable vibration (maximum PWM duty cycle). The ERM vibration motors utilised in the HMIs implementation have a rotation speed range 11,580 (193 Hz) to 15,900 RPM (265 Hz) and vibration amplitude of 0.75 g. Preliminary tests have demonstrated that the particular ERM vibration motors have high inertia and start spinning only at intensities above 30% PWM duty cycle. However, it was also noticed that for most users, only vibration above 50% of maximum intensity was noticeable across the majority of dorsal locations on the hand. Additionally, in SMAM all the intensity values are decreased by 20% in order to bring the overall percept vibration intensity closer to DMAM. The difference in percept vibration intensity is caused by the fact that in SMAM all tactors of the corresponding HMI vibrate simultaneously, whereas in DMAM the total vibration is decomposed to multiple tactors, however, only three tactors can be active at the same time (due to decomposition of vector to axes).

It is anticipated that the introduced system would improve the awareness of the user about the currently planned trajectory of the robot and allow more fluent collaboration. The transparent behaviour of the robot can also lead to increased efficiency in performing tasks since the worker will be informed when blocking the robot from continuing its activity.

### 2.3. Experimental Workspace

**Figure 8:**
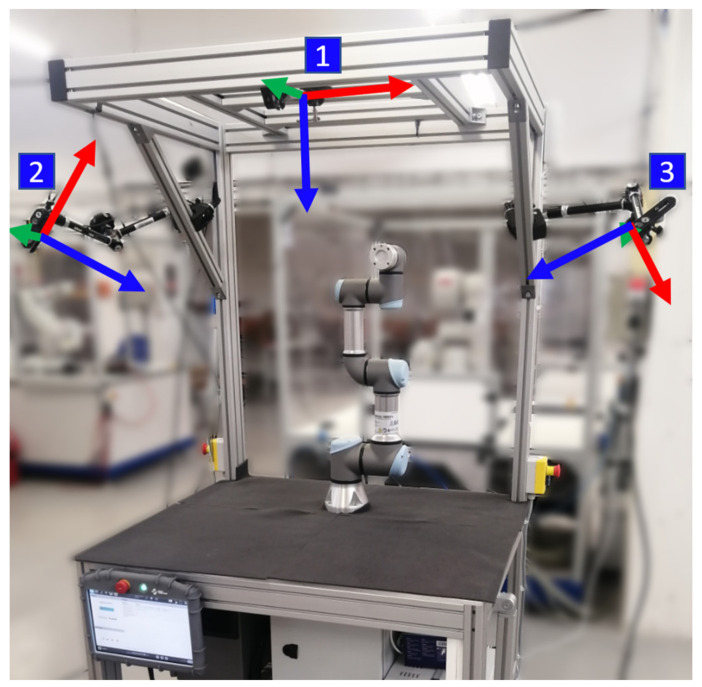
Experimental workspace with depicted locations of three RGB-D sensors (1–3). View directions (Z-axes) are depicted by the blue vectors.

The improved system was tested on an experimental workspace with Universal Robots UR3e collaborative robot (see Figure 8). In order to correctly map the obstacles within the workspace, three RealSense D435 RGB-D cameras are mounted on the workplace frame at different locations. The streaming resolution was set to 424 × 240 at 15 FPS for both RGB and depth data streams, which is considered sufficient for the application. The cameras provide depth images that cover the workspace and allow to map the obstacles (including human body) in the vicinity of the robot.

The control over the system is concentrated into a single PC (laptop HP Omen with Intel i7 2.80 GHz processor and 16 GB of RAM) and utilises the modular architecture offered by ROS (Melodic, Ubuntu 18.04) and divides the software implementation into several separate components.

### 2.4. Improved Hand Tracking Stability

**Figure 9:**
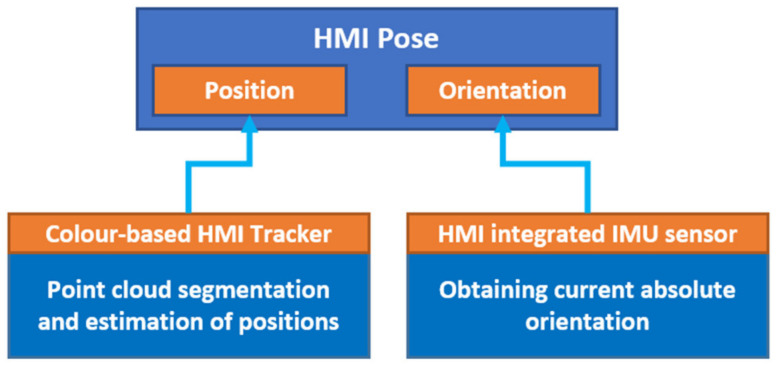
Determining HMI pose.

The task consisting of determining the position and orientation of HMI was divided into two parts (see Figure 9): the relative position of HMIs will be determined using data from depth cameras located at the workplace; the orientation of HMIs will be determined using the IMU sensors integrated into each HMI.

This division is justified by the fact that under the conditions of partial overlap of HMI on the image from the cameras, an accurate determination of its orientation cannot be guaranteed.

**Figure 10:**
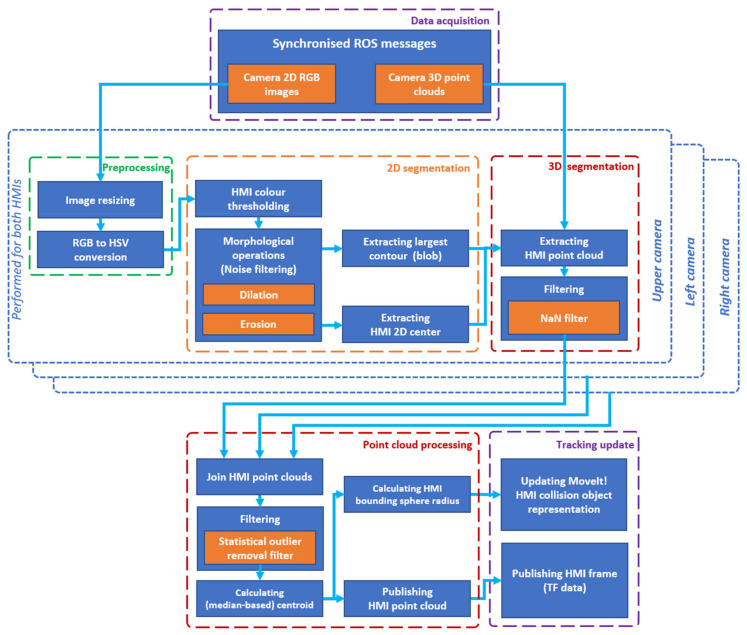
HMI tracker data flow chart.

The task of HMI tracker subsystem is to determine the relative position of HMIs using data from depth cameras located at the workplace. RGB image segmentation is simplified by the fact that the left and right HMIs have distinctive colours (it additionally simplifies the correct determination of the side of the hand at different view angles). We used colour-based point cloud segmentation as a simple alternative to the complex task of detecting a 3D object in a point cloud. A schematic representation of the data processing at this node is displayed in Figure 10.

The improved version of hand tracking utilises both RGB and depth image data from all available cameras in order to provide a stable position output even in the conditions of partial hand surface occlusion on camera images, for example, due to movement of the robot. The colour-based segmentation procedure is performed for data from each camera. The extracted HMI point clouds are then aggregated and filtered together in order to obtain a more stable representation of the positions of HMIs. Found HMI positions are published as ROS transformations. HMIs are internally represented as spherical objects, which allows a fast collision validation.

### 2.5. Motion Planning

The motion replanning subsystem is based on a modified version of ROS MoveIt! [35]. MoveIt! provides robot trajectory planning capability with respect to the current position of obstacles in surrounding space: if the operator precludes the currently planned movement of the robot, the robot is able to replan this movement to avoid collisions with the human operator and continue on the performed activity. The obstacles and the free space is represented in OctoMap [36], which is integrated into MoveIt! The OctoMap is updated with data provided by the depth cameras, which allow to map the obstacles in the workspace of the robot, including parts of the operator’s body.

By default, MoveIt! is supposed to work with static scenes and is not tailored for dynamic environments, where a high reaction speed is a mandatory requirement. In order to obtain better results in the dynamic conditions, multiple aspects of MoveIt! were optimised and changed. The custom version of MoveIt! is based on Melodic distribution (release version 1.0.5). Another task of MoveIt!, in addition to trajectory planning, is to calculate the distance between the HMI and the future trajectory of the robot. MoveIt! internally uses FCL (Fast Collision Library [37]) library to perform discreet collision validation between scene object pairs (5):

(5) $$A\left(c_{A}\right)\cap B\left(c_{B}\right)\neq \emptyset ,$$

where *A*, *B*—collision objects,

$c_{A},\;c_{B}—$ their configurations.

If no overlapping is found, FCL is also able to return the distance between the objects using separation distance query (6):

(6) $$distance=\inf\left\{\|x-y_{2}\|\;:x\;\in \;A\left(c_{A}\right),\;y\;\in \;B\left(c_{B}\right)\right\}$$

The distance can also return the nearest points between an object pair (7):

(7) $$\arg\underset{x\;\in \;A\left(c_{A}\right),\;\;\;y\;\in \;B\left(c_{B}\right)}{\min}\|x-y_{2}\|$$

We utilise this functionality to calculate the collision vector to both HMIs in each moment of the trajectory execution and publish it to other ROS nodes. Each observed HMI is simplistically represented as a spherical collision object. During motion execution, validation of the remaining path is performed at 10 Hz rate. The validation checks collision status between all scene objects (in pairs) at each waypoint of the remaining trajectory segment (see simplified algorithm—Algorithm 1). In addition, for pairs of objects in which one of the objects is an HMI (left or right, never both), the nearest points are found (these points will represent the collision vector).
**Algorithm 1**. Calculation of the collision vectors.
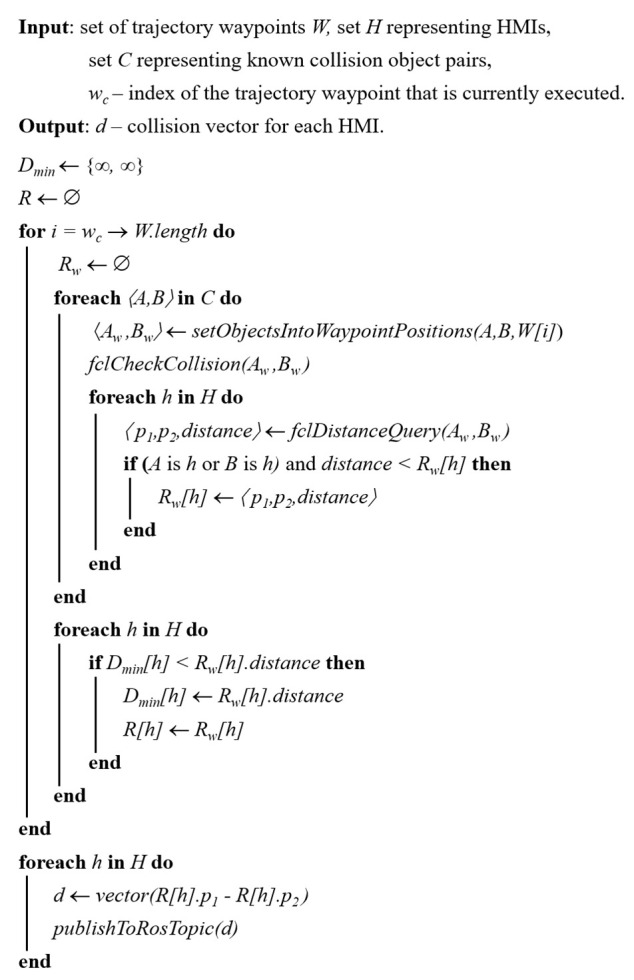


**Figure 11:**
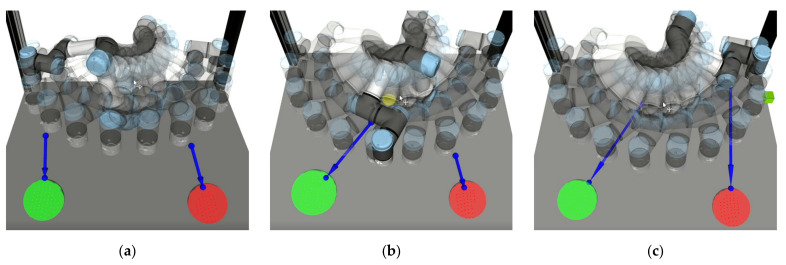
Example of how collision vector changes during a motion sequence: robot moving from left to the right (a–c), collision vector is depicted by a blue arrow, HMIs are represented by green and red spheres.

An example of how calculated collision vector changes during the execution of a motion is illustrated in Figure 11.

## 3. First User Study—Stimuli Differentiation

In order to quantitatively evaluate the ease of differentiating activation of individual tactors of the enhanced haptic notification devices, we created a new experimental task inspired from [27]. The experiment consisted in assessing the reaction of the group of 8 volunteers (test subjects, ranging in age from 25 to 30 years with mean age 26.9 and standard deviation of 1.7, 3 females, 5 males; 5 subjects had previous experience with feedback devices; 6 subjects from a field of expertise other than robotics), whose goal is to accurately recognise the direction communicated by a single HMI glove (six possible directions corresponding to the positive and negative components of XYZ axes, encoded as +X, −X, +Y, etc.) and the intensity of the notification (two possible intensities: maximum and minimum distance notifications, see Table 1, encoded as S—strong notification; W—weak notification).

### 3.1. Experiment Description

**Figure 12:**
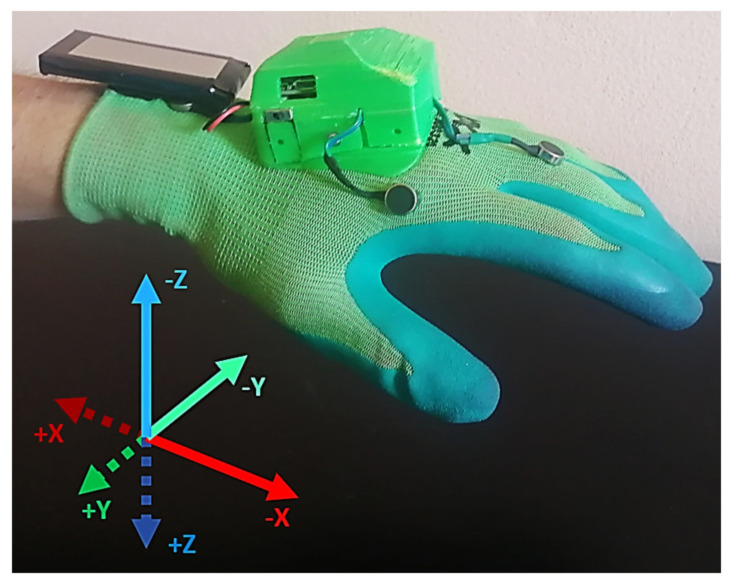
Hand position above the table during the experiment. The participants were instructed to identify the direction communicated by the device by moving the hand in the opposite direction (repulsive prompt) according to tactors’ placement. The movement directions corresponding to the activation of each tactor (+X, −X, +Y, etc.) are visualised as a coordinate frame.

The test subjects were equipped with the HMI on their left hands. Each combination of all directions and both intensities were tested in 5 repetitions (a total of 60 rounds for each volunteer); the order of the tested combinations was random. Before the experiment began, each participant had two trial rounds for testing each possible combination while seeing the correct answer on the screen. Before the start of each round, the volunteer placed the hand with the HMI on the starting position above the table (see Figure 12). Each round started with a 3-2-1 countdown ensuring the volunteer knew the moment when the tactors were activated. The participants were instructed to identify the direction communicated by the device by moving the hand in the opposite direction (repulsive prompt) according to the tactors’ placement. Their task was also to simultaneously report the perceived strength of the vibration (weak or strong) verbally. The reported responses and movement directions were recorded by the experimenter; the vibration was then disabled denoting the end of the round. The volunteers were not allowed to rotate their hand during the trials; however, they could use resting pauses between the trials. The participants were additionally equipped with earplugs in order to avoid hearing the differences in the sounds produced by the tactors during activation with different frequencies.

The necessary safety precautions were taken during all experiments. Participation was not mandatory, and participants could leave any time they chose. All participants reviewed and signed a consent form and read the definition of the experiment before beginning the experiment. After the volunteers agreed to participate, the experimenter clarified the ambiguities regarding the task. 

### 3.2. Results

**Figure 13:**
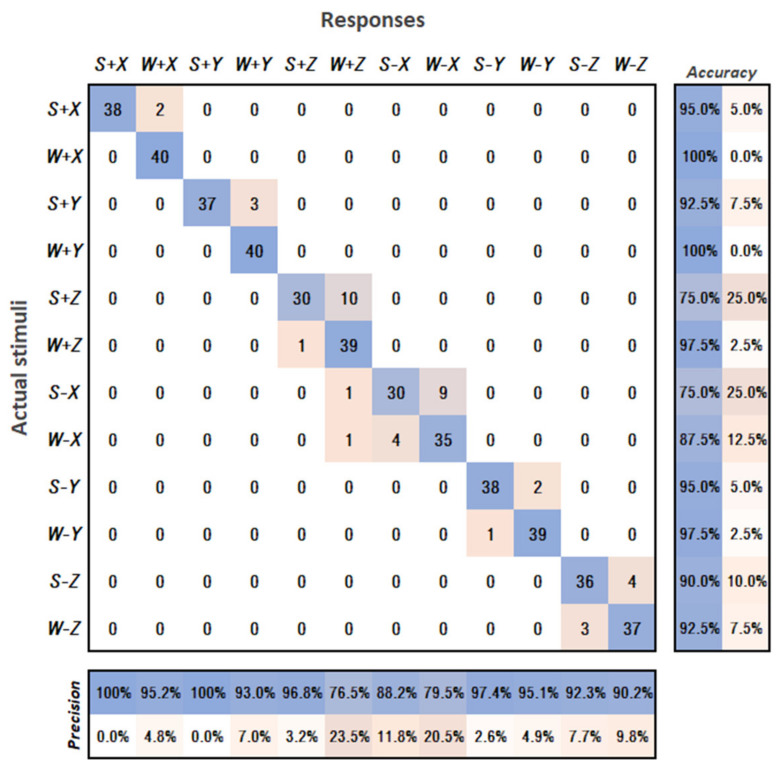
Confusion matrix of all combinations of stimulation intensities (S—strong; W—weak) and directions (+X, −X, +Y, etc.) for all participants. The actual stimuli (ground truth) applied to the participants are pictured along the vertical axis. The participants’ responses are listed along the horizontal axis. Darker values indicate combinations reported with higher frequency. Precision and accuracy values are indicated below and to the right of the confusion matrix, respectively.

Data collected during the experiment included the actual stimuli (direction, intensity) provided to the participant and the response of the participant. The data were processed according to [27] and visualised in the form of a multiclass confusion matrix for all combinations of direction and stimulation intensity, see Figure 13.

The matrix illustrates how the participants confused the provided stimuli. Precision and accuracy were calculated by dividing the total correct stimuli identification, where the stimuli and response are the same (diagonal entry for that response), by the total instances where that response was given (the column or row sum for that response).

According to the data, during all rounds of the experiment, the participants were unable to correctly recognise the activated tactors −X and +Z only twice. In both cases, the regarded participants were then able to correctly recognise the activated tactors after the glove position was adjusted (folds of the glove fabric caused shifts of the tactors). Apart from this, it can be noticed that the same tactors had the lowest accuracy in determining the vibration intensity (strong or weak) among users, whereas differentiation for other tactors was substantially more accurate. Most often, participants confused strong activation with weak activation; less often the opposite situation arose. In most cases, the test subjects were able to successfully recognise the same activations in the next rounds after being corrected by the experimenter once. Some test subjects reported that the strong and weak stimuli were perceived differently when produced by different tactors. Additionally, both −X and +Z tactors are located at the dorsal side of the hand (see Figure 5). The misperceptions can be partially explained by the differences in the sensitivity and spatial resolution of the different areas of the skin around the hand, which has been reported by several studies [38,39]. As such, in future, more research is needed to find the optimal placement of tactors along with the optimal vibration intensity of notifications.

## 4. Second User Study—Trajectory Awareness

In order to evaluate the usability of the developed notification mode for the haptic devices, we adopted the experimental task from the previous work [20]. The experiment consisted in assessing the reaction of the group of 17 volunteers (test subjects, ranging in age from 23 to 40 years with mean age 26.4 and standard deviation of 4.09, 2 females, 15 males; 14 subjects had previous experience with feedback devices; 5 subjects from a field of expertise other than robotics), whose goal is to accurately recognise the goal position of the robot during its movement. Data collected during the experiment included objective (measured parameters of each testing cycle) and subjective (survey-based) parameters. Data analysis further allowed us to compare the interfaces and evaluate the research hypotheses.

### 4.1. Experiment Description

**Figure 14:**
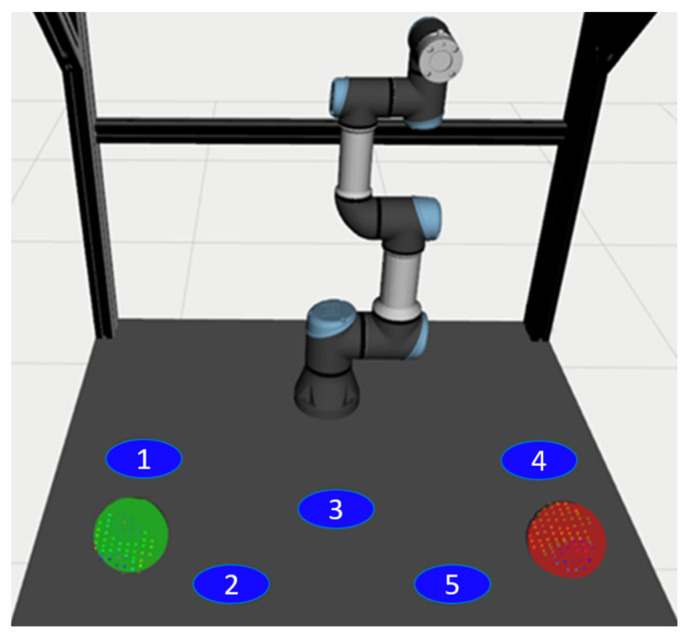
Preview of the workplace and the robot’s goal positions (1–5); hand positions are marked with red (right hand) and green (left hand) spheres; the robot is in the initial position.

The experimental task was based on the idea of sorting parts into different containers, where it is not known in advance in which container the robot will have to place the next part. In each round of the experiment, the robot planned its trajectory from its starting position (same for all trials) to one of five possible TCP goal positions (see Figure 14). Each goal position was marked and labelled on the table for the real workspace. At the end of the trials, each volunteer filled in the task-specific questionnaire consisting of questions related to the usability of each interface and the perceived naturalness during the task execution.

During the experiment, each volunteer tested the following variations of completing the task:

- V1—Without HMI: the volunteer is not equipped with HMIs and has no feedback on approaching the robot’s future trajectory. Only visual information is available for determining the goal position of the robot (the volunteer sees the whole robot).
- V2—Equipped HMIs in SMAM: the volunteer is equipped with HMIs on both hands. The volunteer can use the HMI feedback (when moving hands) to determine the goal position of the robot. The volunteer is also instructed to watch their hands instead of watching the robot.
- V3—Equipped HMIs in DMAM: the volunteer is equipped with HMIs on both hands. Activation of the vibration motors depends on the relative orientation between the collision vector and the corresponding HMI. The volunteer can use the HMI feedback (when moving hands) to determine the goal position of the robot. The volunteer is instructed to watch their hands instead of watching the robot.

In all cases, the volunteers did not see a visualisation of the trajectory on the monitor.

The necessary safety precautions were taken during all the pilot experiments, and all the test subjects were informed about the potential risks and behaviour in safety-critical situations. Participation was not mandatory, and participants could leave any time they chose. All participants reviewed and signed a consent form and read the definition of the experiment before beginning the experiment. After the volunteers agreed to participate, the experimenter clarified the ambiguities regarding the task and the principles of each interface. In addition, before the start of the experiment, each volunteer was shown the movements of the robot along standard trajectories to all five targets. During the experiment run no harm was done to the volunteers.

Each round started with a 3-2-1 countdown ensuring the volunteer knew the moment when the robot started to move. During movement to the random goal position, the robot TCP speed was limited to the maximum value of 80 mm/s. The task of the volunteers was to determine the target position to which the robot is currently heading and, at the same time, avoid colliding with the robot. If the goal position number reported by the volunteer was correct, the task completion time was recorded, and the attempt was counted as successful. In case the reported goal position number was incorrect, the volunteer intervened in the planned trajectory of the robot or failed to determine the robot’s goal during movement of the robot, the final result of the attempt was recorded as unsuccessful, and the measured time was discarded. The objective parameters (time, success rate) of each attempt were recorded. If the user had reported the guessed goal position before the robot reached it, the person responsible for carrying out the experiment might have interrupted the movement of the robot and moved it back to the initial position to spare the time of the experiment.

Testing of each interface option was performed in five rounds, i.e., a total of 15 rounds for each volunteer. Before testing a specific interface, the volunteer had three trial rounds. The order of the tested interfaces (V1, V2, V3) was selected at random for each volunteer to mitigate the order effect on the measured parameters. In each round, the robot started its movement from the same starting position (arm vertically straightened above the worktable, see Figure 14), and the goal position of the robot was selected randomly (i.e., the randomly selected goal positions may have repeated multiple times). Before the start of each round, the volunteer placed their hands on the starting positions marked in Figure 14; these positions were selected so that the user in the starting position did not preclude the robot in any of the target positions. During each trial, the users were free to move their hands across the workspace (as long as it did not cause the robot to replan its motion). At the end of all rounds, the volunteer filled in the questionnaire.

### 4.2. Hypotheses

The initial hypotheses are based on the suggestion that the improved feedback system should enhance the awareness of the human operator about the boundaries of the space where the robot will perform the future movement. It is anticipated that DMAM will decrease the time needed to complete the task and the rate of successful task completion. The dependent measures (objective dependent variables) were defined as task completion time and task success rate. The within-subjects independent variable was defined as a robot intent–communication interface (V1, V2, V3).

Overall, it is expected that the test subjects will perform better (lower task completion times, higher task completion rate) and will have higher subjective ratings when equipped with HMIs. It is also anticipated that the intuitive spatial feedback in DMAM will allow them to achieve the best results. The experimental hypotheses were defined as follows:

- H1.1: The efficiency of the test subjects will be greater with equipped HMIs (in both modes: V2, V3) than without HMIs. Higher efficiency is categorised as lower task completion time. This hypothesis is based on the suggestion that both HMI modes contribute to task performance.
- H1.2: The efficiency of the test subjects will be greater with equipped HMIs with directional feedback enabled (V3) than with equipped HMIs in simultaneous motor activation mode (V2). Higher efficiency is categorised as lower task completion time. This hypothesis is based on the suggestion that the directional haptic feedback significantly contributes to the user awareness about the future trajectory of the robot.
- H1.3: Time taken by each test subject to correctly determine the robot’s goal position will be similar during all rounds when equipped with HMIs (V2, V3). In contrast, there will be high variation in task completion times between the rounds when the determination will be based solely on the available visual information. To test this hypothesis, task completion time will be measured for each subject in each task condition, and the standard deviation of the measurements will be compared. This hypothesis is based on the suggestion that users’ awareness enhanced by the haptic feedback is not influenced by the differences in the trajectories, whereas in the case of visual feedback, the awareness is dependent on the actual trajectory shape.
- H2: The task efficacy of the test subjects will be greater when equipped with V3 compared to V2 interface. This hypothesis is based on the suggestion that the additional information provided by the directional feedback will be more intuitive and comprehensive. However, it is also suggested that the success rates of V1 and V3 interfaces will be similar, as it is assumed that V3 provides enough information for the users to recognise the goal position of the robot even though they will not explicitly observe the robot’s trajectory.
- H3.1: Volunteers will subjectively percept the task as simpler when performing the tasks equipped with HMIs (V2, V3) than by relying solely on the available visual information (V1). This hypothesis is based on the suggestion that the haptic feedback significantly contributes to the user awareness about the future trajectory of the robot, thus making the task cognitively easier.
- H3.2: Volunteers will subjectively percept the task as simpler when performing the tasks when equipped with HMI in directional feedback mode (V3) than with the other two interfaces (V1, V2). This hypothesis is based on the suggestion that the directional haptic feedback significantly contributes to the user awareness about the future trajectory of the robot, thus making the task cognitively easier.
- H3.3: Volunteers will report better subjective ratings when performing the tasks when equipped with HMI in directional feedback mode (V3) than in the case of HMI without directional feedback (V2). This hypothesis is based on the suggestion that the directional haptic feedback further improves the user awareness about the future trajectory of the robot.

Efficiency was defined as the time taken by the human subjects to complete the task, and effectiveness was defined as the percentage of successful task completion. Efficiency and effectiveness were evaluated objectively by measuring these parameters for each test subject during rounds of the experiment. Apart from objective parameters, multiple subjective aspects of interacting with different types of interfaces were mapped. The analysis of subjective findings was based on responses to 33 questions. The main points of interest focused on the understanding of the robot’s goals and motions, the feel of security when working closely with the robot, ergonomics, and the overall task difficulty. For both collaboration approaches, the participants were asked to indicate on a 1–5 Likert scale (scaling from 1—“totally disagree” to 5—“totally agree”) the extent to which they agreed with the defined statements.

The first four questions (Q1–Q4, see Table 2) were task- and awareness-related and aimed to map the comparative aspects of the collaboration during the task execution with all tested interface variants V1, V2 and to test the general clarity of the provided instructions.

The questions defined in Table 3 were additionally defined for HMI interfaces.

Finally, for the HMI with directional feedback (V3), an additional three questions were defined to investigate the clarity and usability of the directional feedback—Table 4.

The test subjects also could choose a single interface of their own preference. The volunteers could additionally leave a free comment about any topic related to each interface option. To minimise the effect of bias (practice effect [40]) caused by the order in which participants interacted with each interface, the order was chosen randomly for each participant during the experiment. To determine whether the differences between objective measures in the three conditions (V1, V2, V3) were significant at the 95% confidence level, the repeated measures analysis of variance (ANOVA) was applied.

### 4.3. Results—Objective Data Evaluation

**Figure 15:**
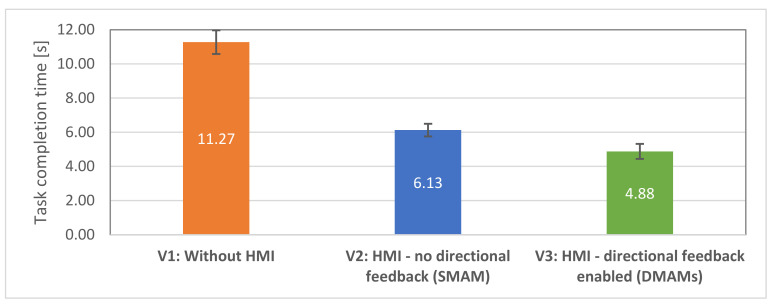
Average task completion time with standard errors for all 17 participants: lower is better.

Hypothesis H1.1 states that the efficiency of the human–robot collaborative team will be higher in the case of the equipped HMIs (V1, V2). The total time taken for completing the tasks was measured and compared between the three conditions. It can be seen from Figure 15 that the average task completion time is significantly higher in V1 condition than V2 and V3 conditions. Task completion time was found to be lower in V2 and the lowest in V3. The completion time means were compared using ANOVA test, and statistically significant differences were found, F (2,32) = 50.47; *p* < 0.00001.

The following post-hoc *t*-tests revealed statistically significant difference in mean task completion times between V1 (M = 11.27, SE = 0.69) and V2 (M = 6.13, SE = 0.37) interfaces, t (16) = 6.77, *p* < 0.00001. There was also a significant difference in mean task completion times between V1 and V3 (M = 4.88, SE = 0.44) interfaces, t(15) = 8.32, *p* < 0.00001. Thus, hypothesis H1.1 was supported.

Hypothesis H1.2. stated that the test subjects will have higher efficiency when with equipped V3 interface comparing to V2 interface. Post-hoc *t*-tests revealed a statistically significant difference in mean task completion times between V2 and V3, t (16) = 2.81, *p* < 0.01. Thus, hypothesis H1.2 was supported.

**Figure 16:**
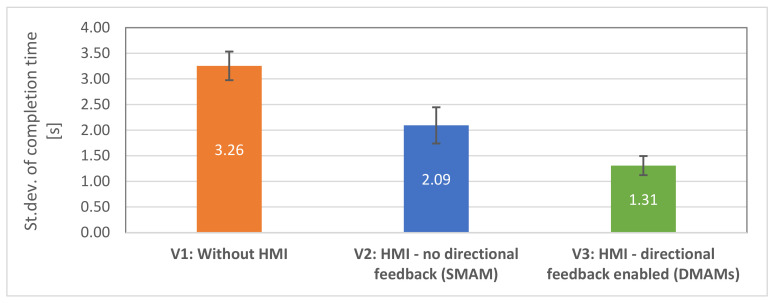
Standard deviations of the task completion time: lower is better.

Hypothesis H1.3 stated that the time taken by test subjects to correctly determine the goal position will be similar in all rounds when equipped with HMIs (V2, V3). To investigate the hypothesis, the task completion time for all test subjects was analysed using the standard deviations. It was observed that the standard errors for each subject for both HMI modes (V2: M = 2.09, SE = 0.35; V3: M = 1.31, SE = 0.19) were significantly lower than for visual inspection (V1: M = 3.26, SE = 0.28), implying that these modes allowed the participants to perform the task within a similar amount of time, whereas the time need with V1 interface was highly different in each round—see Figure 16. The deviations were compared using ANOVA test, and statistically significant differences were found, F (2,32) = 11.31; *p* < 0.001. In contrast, standard errors in V1 conditions were comparatively higher (post-hoc *t*-tests with Bonferroni correction α/2, V1–V2: t (16) = 2.49, *p* = 0.12, V1–V3: t (16) = 5.01, *p* < 0.0001. This suggests that the provided haptic feedback was intuitive and took approximately the same amount of time for the participants in each round to determine the goal position, thus supporting the H1.3 hypothesis.

**Figure 17:**
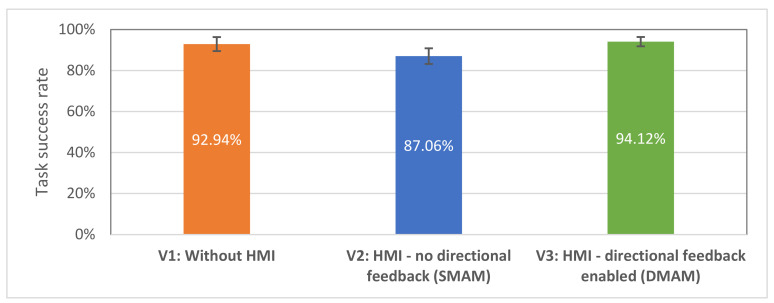
Average task success rate for all 17 participants: higher is better.

Hypothesis H2 states that the effectiveness of the human–robot collaborative team will be similar in all three conditions. The average task success rates with standard errors are shown in Figure 17.

The task success rates were compared using ANOVA test. Average success rates were lower for V2 interface (M = 87.06%, SE = 3.81%) comparing to V1 (M = 92.94%, SE = 3.40%) and V3 (M = 94.12%, SE = 2.28%), this supports the H2 hypothesis; however, no statistically significant differences were found, F (2,32) = 1.92; *p* = 1.63. One of the factors that may have led to a lower success rate with V2 interface may be that the haptic feedback (V2) did not provide the test subjects with enough spatial information, which led to an ambiguous determination of the goal positions (since V2 interface did not provide any information about the closest collision vector to the robot’s trajectory). That factor will be further covered during the analysis of the subjective responses.

In general, the objective findings indicate that both HMI modes allowed users to achieve better results during the test. Additionally, V3 interface with directional feedback allowed the test subjects to achieve the best task completion times.

### 4.4. Results—Subjective Data Evaluation

**Figure 18:**
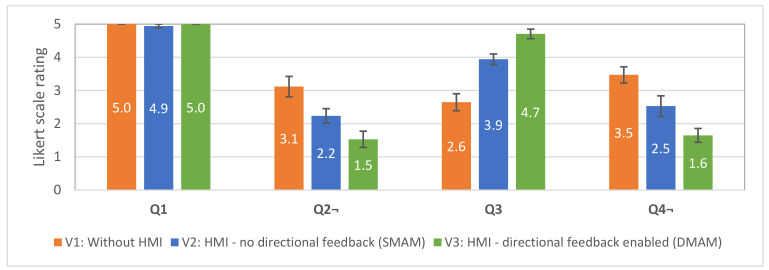
Average scores with standard errors for the questions Q1–Q4 used in the user study. Score 5 denotes “totally agree” and 1—“totally disagree”. Questions Q1, Q3—higher is better; Q2, Q4—lower is better.

All 17 participants answered, in total, 33 template questions, and the results were analysed. The first four questions (Q1–Q4) were common for all interfaces and intended to capture the differences in subjective usability between these interfaces. The average scores with the standard errors are shown in Figure 18. To determine whether the differences between ratings for each questionnaire item between test conditions (V1, V2, V3) were significant at the 95% confidence level, the repeated-measures analysis of variance (ANOVA) was applied.

Q1 was intended as an indicator of clarity of the task between the test subjects. The results show that the test subjects understood the provided instructions (there are no statistically significant differences in the responses in all three conditions). Q2 question was related to the subjective perception of the task difficulty in each condition. Statistically significant differences were found, F (2,32) = 13.07; *p* < 0.0001. The following post-hoc *t*-tests revealed statistically significant difference in responses between V1 (M = 3.12, SE = 0.31) and V2 (M = 2.24, SE = 0.22) interfaces, t (16) = 2.86, *p* < 0.01. There was also a significant difference in responses between V1 and V3 (M = 1.53, SE = 0.24) interfaces, t (16) = 4.62, *p* < 0.001. V2 performed better than V1; V3 performed better than V1 and V2. Q3 question intended to compare the subjectively perceived improvement in the awareness related to the robot’s trajectory. Statistically significant differences were found, F (2,32) = 29.69; *p* < 0.00001. The following post-hoc *t*-tests revealed statistically significant difference in responses between V1 (M = 2.65, SE = 0.26) and V2 (M = 3.94, SE = 0.16) interfaces, t (16) = 4.22, *p* < 0.001. There was also a significant difference in responses between V1 and V3 (M = 4.71, SE = 0.14) interfaces, t (16) = 7.42, *p* < 0.00001. V2 performed better than V1; V3 performed better than V1 and V2. Q4 question was designed to assess whether the participants subjectively felt the need for additional information in order to reliably recognise the trajectory of the robot. Statistically significant differences were found, F (2,32) = 16.71; *p* < 0.0001. The following post-hoc *t*-tests revealed statistically significant difference in responses between V1 (M = 3.47, SE = 0.24) and V2 (M = 2.53, SE = 0.31) interfaces, t (16) = 3.34, *p* < 0.01. There was also a significant difference in responses between V1 and V3 (M = 1.65, SE = 0.21) interfaces, t (16) = 5.45, *p* < 0.0001. V2 performed better than V1; V3 performed better than V1 and V2. Thus, hypotheses H3.1 and H3.2 are supported by the responses to Q2–Q3. The subjective findings are also supported by the evaluation of the objective performance of the test subjects.

**Figure 19:**
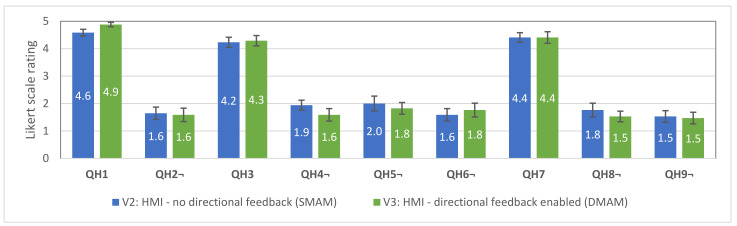
Average scores with standard errors for the questions QH1–QH9 used for evaluation of the HMI usability. Score 5 denotes “totally agree”, and 1—“totally disagree”. Questions QH1, QH3, QH7—higher is better; QH2, QH4, QH5, QH6, QH8, QH9—lower is better.

Next, subjective responses to QH1–QH9 were analysed (see Figure 19). These questions are intended to compare the subjective usability of the two HMI modes.

Only minor differences in users’ responses were noticed with the largest ones being QH1 (awareness about the trajectory), QH4 (clarity of haptic feedback), QH5 and QH6 (strength of the vibration), QH8 (perception load); however, statistically significant differences between the scores was found only in QH1 (V2: M = 4.59, SE = 0.12; V3: M = 4.88, SE = 0.08; t (16) = 2.06, *p* = 0.28) and QH6 (V2: M = 1.59, SE = 0.23; V3: M = 1.76, SE = 0.25; t (16) = 1.85, *p* = 0.04), favouring the V3 interface. Thus, hypothesis H3.3 is not supported. In general, according to the responses, both interfaces performed well. In both cases (V2, V3) there were few participants who reported that the vibration feedback was too strong and participants who reported feedback as too weak, thus leading to a conclusion that the optimal intensity of vibration feedback should be further investigated or should allow personal adjustments.

**Figure 20:**
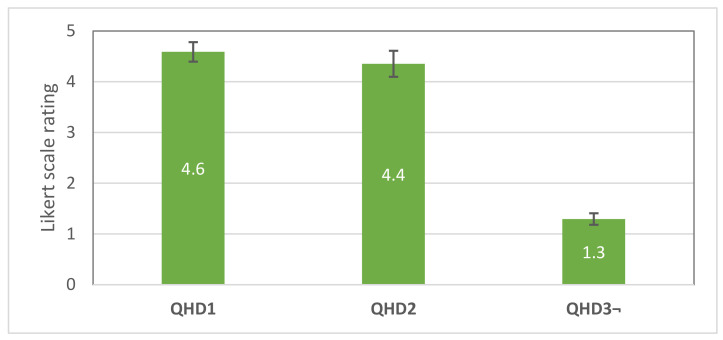
Average scores with standard errors for the questions QHD1–QHD3 used for evaluation of the usability of V3 interface. Score 5 denotes “totally agree”, and 1—“totally disagree”. Questions QHD1, QHD2—higher is better; QHD3—lower is better.

Responses to questions QHD1–QHD3 (see Figure 20) were used to analyse the general attitude of the test subjects toward the directional feedback provided by V3 interface.

Subjective responses show that the users were generally satisfied with provided feedback and the notifications were clear for them. The users could also choose the one interface of their personal preference. The vast majority of the participants (82.35%) favoured the V3 (HMI with directional feedback) over other interfaces. Most of the participants also mentioned the V3 as more intuitive comparing with V2.

The user survey additionally offered to leave a free comment for each of the tested interfaces. Major themes mentioned in the comments included user perceptions of the haptic feedback. For example, three subjects noted that with the V3 interface, they could not simultaneously recognise the indicated direction and intensity since the change in the orientation of the hand also manifested itself in a change in the vibration intensity. The same users also noted that during directional activation of 2 or 3 adjacent motors, it was difficult for them to decide which one vibrated the most. For these participants, the V2 interface was simpler, as it allowed them to perceive the change in intensity in several places around the arm. Some subjects noted that the vibration intensity was perceived differently in different places around the arm (for example, the vibration of the tactor +Z was less percept by the volunteers). This suggests that additional research is needed to find the optimal arrangement of vibration motors while maintaining their spatial configuration (which currently represents an orthogonal coordinate system). Multiple test subjects mentioned that the size of the work gloves was not optimal. This issue may be solved by implementing HMI in the form of modules that will be locked onto the universal work gloves.

Based on the analysis of the subjective responses and free comments from the studies, V3 was favoured by users with regard to intuitiveness, clarity, and feedback. During the user study, it was noted that the significant difference in the task completion time between V2, V3 was mainly due to the fact that V3 interface offered the users additional information about the direction of a potential collision, allowing them to complete the task almost immediately (up to 2s) in some instances. Unlike V3, V2 interface required the users to use both their hands to more quickly map the gradient of vibration intensify in the ambiguity cases (pairs of goal positions 1, 2, and 4, 5).

## 5. Discussion

The evaluation of the results of the first user study demonstrated that, in general, the current locations of the tactors in HMIs enable the users to accurately recognise activations of the individual tactors. During the test, only two incorrect reports were obtained related to the position of the tactors, which can potentially be explained by the relatively large distance between the individual tactors. Nevertheless, the results also allowed to identify tactors for which vibration intensity was most often confused by users. The misperceptions can be partially explained by the differences in the sensitivity of the different areas of the skin around the hand. As such, more research is needed to find the optimal placement of tactors along with the optimal vibration intensity of notifications.

The evaluation of the objective parameters measured during the second user study showed significant evidence supporting hypotheses H1.1, H1.2, H1.3. The participants took less time to recognise the goal position when equipped with HMIs. The time required by test subjects to successfully complete the task with HMIs equipped had low variation (unlike with visual feedback), potentially indicating that user awareness was independent of the shape of the robot movement. The overall task success rate was higher for HMI in DMAM comparing to SMAM.

The subjective findings from the structured and free response questions of the second user study supported hypotheses H3.1, H3.2, which stated that participants would be more satisfied with HMI interface compared to baseline. Overall, participants favoured the HMI with regard to human–robot fluency, clarity, and feedback.

**Figure 21:**
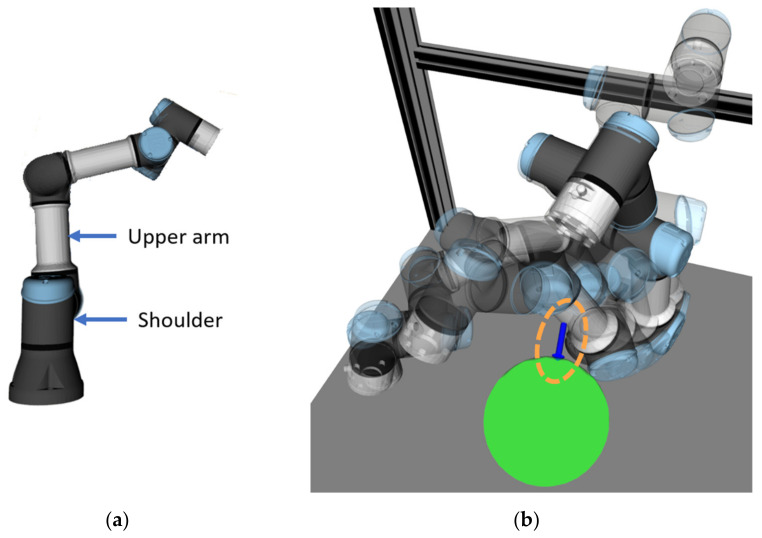
Collision vector computed during movement of the robot to the first goal position: (**a**) names of the related robot links; (**b**) collision vector starting from upper arm of the robot.

During the tests, another factor related to tactile feedback was discovered: in some cases, the participants could not correctly recognise the position of the target because the calculated collision vector was drawn not from the flange of the robot (as users expected) but from the upper arm link of the robot since this part was closer to the HMI during the planned movement (see Figure 21). A possible solution is to set the reaction distance for each robot link separately, depending on the danger that they present. For example, reaction distance can be lower for the upper arm and shoulder links of the robot since they move more slowly. The reaction distance can also be set dynamically depending on the actual speed of the corresponding links. A similar approach was implemented in the work of Scalera et al. [41], where the actual size of the virtual safety zone for each robot link was set dynamically according to its speed and related safety standards.

Taken together, the results of the experiments indicate the usefulness of the developed system since it improves user’s awareness about the motion plan of the robot. While not conclusive, these results indicate the potential of a haptic feedback-based approach in improving the interaction quality in human–robot collaboration.

## 6. Conclusions

In this work, we proposed an improvement of the tactile feedback system for notification of the user about approaching the future trajectory of the robot. The system combines active collision avoidance of the collaborative robot with hand-worn tactile notification devices, which alerts the human worker when approaching the space that will be occupied by the robot during its future trajectory: the closer the worker’s hand approaches the future segment of the trajectory, the stronger the vibration provided by the device.

The improved system extends the concept of an improved human–robot collaboration presented in the previous work by adding orientation-dependency to the provided feedback. Each notification device is equipped with six tactors spatially organised into an orthogonal coordinate system, where each tactor represents a single direction of one axis. An arbitrary vector in this configuration is represented by the activation of multiple adjacent motors with different vibration intensity, depending on the device orientation. It was expected that the enhanced notification system will further improve the intuitiveness of the information provided to the users. Additionally, the system’s reliability was significantly improved by implementing an accurate hand tracking system that combines the data from all available RGB-D sensors.

A prototype of the improved notification system was implemented and tested in two user studies. The first study aimed to quantify the ease of differentiating activation of individual tactors by HMI users. The collected data allowed to identify the tactors for which vibration intensity was most often confused by users. This information will be used in the future to optimise the HMI design.

The second user study aimed to assess the overall usability of the enhanced notification mode for improving the operator’s awareness about the planned trajectory of a collaborative robot in a shared environment. The task of the participants was to accurately recognise the goal position of the robot during its movement. Data collected during the second user study included both objective parameters and subjective responses of the participants. Statistical data analysis allowed to compare the interfaces and evaluate the stated research hypotheses. The implemented spatial tactile feedback allowed the participants to complete the task faster compared to both the baseline and the initial version of the haptic interface. The majority of the users also favoured the new feedback mode for its intuitiveness and clarity. Based on the user feedback and notes taken during the experiment, multiple new aspects of the system usability were revealed. Taken together, the results indicated the potential of the developed haptic feedback-based approach for improving the interaction quality in human–robot collaboration.

Future research will focus on the improvement of the notifications provided to the user. The first direction is the improvement of the feedback device implementation. Due to the difference in the sensitivity of skin regions around the hand, more research is required to find optimal placement for the tactors since the perceived vibration intensity may vary across their placement, leading to misperceptions of the provided stimulus. The second direction of the improvement is adjusting the rules by which notifications are provided, making them even more intuitive and less disruptive during long work. However, finding optimal values that will be sufficiently perceptible and convenient for all users requires standalone research and multiple user studies since the vibration intensities must be designed with regard to both individual user perception and safety standard ISO 5349-1, which defines the measurement and evaluation of exposure to hand-transmitted vibration.

## Figures and Tables

**Table 1:** Notification vibration intensities.

Notification	Vibration Intensity	Directional	Duration	Description
Inaccessible goal notification	85%	No	Continuous	The robot was not able to find a feasible path to the goal
Replanning notification	95%	No	0.3 s	The robot has replanned its motion in order to avoid a collision
Distance notification (maximum)	80%	Yes	Continuous	The user’s hand is about to block the currently planned robot trajectory
Distance notification (minimum)	60%	Yes	Continuous	The user’s hand is approaching the future segment of the robot’s trajectory

**Table 2:** General questions for all the tested interfaces.

General Question
Q1. The task was clear for me
Q2. The task was demanding
Q3. It was simple to determine the goal position of the robot
Q4. More information was needed to accurately determine goal position of the robot

**Table 3:** HMI-related questions.

HMI-Related Questions
QH1. HMI improved my awareness of the robot’s future trajectory
QH2. Work with HMI required long training
QH3. Work with HMI improved my confidence in safety during the task
QH4. Haptic feedback (vibration) from HMI was misleading
QH5. Haptic feedback (vibration) from HMI was too strong
QH6. Haptic feedback (vibration) from HMI was too weak
QH7. Haptic feedback (vibration) from HMI was sufficient
QH8. Haptic feedback (vibration) from HMI overwhelmed my perceptions
QH9. Use of HMI was inconvenient or caused unpleasant sensations during activation

**Table 4:** Questions related to HMI with directional feedback (DMAM).

HMI DMAM-Related Questions
QHD1. Haptic feedback (vibration) from HMI about the expected direction of the collision was clear for me
QHD2. Haptic feedback (vibration) from HMI about the expected direction of the collision captured the real trajectory of the robot correctly
QHD3. I was not able to determine the direction of the haptic feedback

## Data Availability

The data presented in this study are available on request from the corresponding author. The data are not publicly available due to project restrictions.
